# B-mode ultrasound diagnostic flowchart for solid breast masses: JABTS BC-01 study

**DOI:** 10.1007/s10396-020-01072-0

**Published:** 2021-01-03

**Authors:** Takanori Watanabe, Takuhiro Yamaguchi, Eriko Tohno, Hiroko Tsunoda, Setsuko Kaoku, Kanako Ban, Ryoji Watanabe, Takeshi Umemoto, Koichi Hirokaga, Toshikazu Ito

**Affiliations:** 1 Department of Breast Surgery, National Hospital Organization Sendai Medical Center, 2-11-12 Miyaginohara, Miyagino-ku, Sendai, Miyagi 983-8520 Japan; 2 Division of Biostatistics, Tohoku University Graduate School of Medicine, 1-1 Seiryo-machi, Aoba-ku, Sendai, Miyagi 980-8574 Japan; 3 Tsukuba International Breast Clinic, 2F Tsukuba CITYIA Bldg., 2-8-8 Azuma, Tsukuba, Ibaraki 305-0031 Japan; 4 Department of Radiology Diagnostic Breast Imaging, St Luke’s International Hospital, 9-1 Akashi-cho, Chuo-ku, Tokyo, 104-8560 Japan; 5 Department of Ultrasonics, National Hospital Organization Osaka National Hospital, 2-1-14 Hoenzaka, Chuo-ku, Osaka, Osaka 540-0006 Japan; 6 Department of Cancer Detection and Diagnosis, Tokyo Health Service Association, 1-2 Ichigaya-sadohara-cho, Shinjuku-ku, Tokyo 162-8402 Japan; 7 Department of Breast Center, Itoshima Medical Association Hospital, 532-1 Urashi, Itoshima, Fukuoka 819-1112 Japan; 8 Department of Senology, Moriya Keiyu Hospital, 980-1 Tatsuzawa, Moriya, Ibaraki 302-0118 Japan; 9 Department of Breast Surgery, Hyogo Cancer Center, 13-70 Kitaoji, Akashi, Hyogo 673-8558 Japan; 10 Department of Surgery, Faculty of Medicine, Kindai University, 377-2 Ohno-Higashi, Osaka-Sayama, Osaka 589-8511 Japan

**Keywords:** Breast ultrasound, Breast cancer, Diagnostic criteria, Multivariate analysis, Multicenter study

## Abstract

**Purpose:**

Breast ultrasound has been widely used as an essential examination for diagnosing breast cancer. However, standardized diagnostic criteria are as yet lacking. This study aimed to develop a simple diagnostic flowchart for beginners learning breast ultrasonography. The diagnostic flowchart was developed based on the recall criteria widely used in Japan.

**Methods:**

We conducted a multicenter study to examine recall criteria usefulness in the diagnostic phase of breast disease. Women with ultrasound-visible breast masses who underwent B-mode breast ultrasound examination were recruited from 22 hospitals in Japan between September 2009 and January 2010. B-mode images were evaluated by members of the centralized image interpretation committee. We developed the new diagnostic flowchart based on the results. The usefulness of the diagnostic flowchart was assessed by employing datasets from the current study and another study which we conducted (BC-04 study).

**Results:**

We evaluated 1045 solid masses (malignant: 495, benign: 550). Multivariate analysis showed that shape, margin, echogenic halo, interruption of the mammary gland interface, and depth width ratio were significant findings for distinguishing between benign and malignant masses. We modified the recall criteria and developed our novel diagnostic flowchart using these findings. The sensitivity and specificity of the new flowchart (current study: 0.97, 0.45; BC-04 study dataset: 0.95, 0.45) were similar to those of experts (current study: 0.96, 0.54; BC-04 study dataset: 0.98, 0.38).

**Conclusion:**

We developed a simple diagnostic flowchart for breast ultrasound. This flowchart is anticipated to be applicable to educating beginners learning breast ultrasound.

## Introduction

Breast ultrasound has improved remarkably due to advances in imaging technologies, such as tissue harmonic imaging, spatial compounding, Doppler ultrasound, and elastography. These advances have improved the ability to make an accurate differential diagnosis between benign and malignant lesions. However, diagnostic criteria for breast masses have yet to be standardized.

Breast ultrasound and mammography have been widely used as essential examinations for diagnosing breast cancer in Japan. The Japan Association of Breast and Thyroid Sonology (JABTS) was established in 1998. In 2004, JABTS published the Guidelines for Breast Ultrasound Diagnosis (1st edition) [1]. In the Guidelines, we proposed a diagnostic flowchart for breast masses using the recall criteria for ultrasound breast cancer screening. This flowchart and the criteria were developed based on the opinions of breast ultrasound experts. The recall criteria are very simple, and were developed for breast ultrasound screening. The diagnostic flowchart was developed for breast ultrasound diagnosis. However, the diagnostic flowchart was complex and difficult to remember. As a result, the diagnostic flowchart is not now in widespread use in Japan, although the recall criteria are widely used. There is, however, a problem with using the recall criteria for diagnosing breast masses. Since biopsy is performed to confirm the final diagnosis of breast cancer, it is important to decide whether to recommend a biopsy or observation at the time of breast ultrasound diagnosis. The recall criteria are not applicable to making this judgment. Therefore, we cannot use the recall criteria for this purpose. The aim of this study was to develop a simple new diagnostic flowchart for solid breast masses to facilitate the decision as to whether biopsy or observation should be recommended. We conducted a multicenter study and obtained findings useful for distinguishing between benign and malignant masses. Based on the results, we developed the novel diagnostic flowchart presented herein. To evaluate the usefulness of this new diagnostic flowchart, we employed a patient dataset from another of our multicenter studies in addition to the data obtained in this study.

## Materials and methods

### Data collection

Women with an ultrasound-visible breast mass who underwent B-mode breast ultrasound examination were recruited from 22 hospitals in Japan between September 2009 and January 2010. Ultrasound units with linear transducers exceeding 10 MHz were used in this study. Exclusion criteria were as follows: 1. simple cysts, 2. lesions already being followed by ultrasound, 3. lesions subjected to vacuum-assisted biopsy at another hospital, 4. masses larger than 5 cm in maximum diameter. Biopsy or observation was selected according to the routine clinical practices of each hospital. Lesions with no significant change during the 2 years of observation were regarded as being benign in this study. Static B-mode digital images and histopathological data without personal information were collected at the clinical research data center at Tohoku University Hospital.

### Informed consent

The institutional review board or the ethics committee at each hospital approved this prospective observational study. Written informed consent was not required in this trial according to the ethical guidelines for epidemiological research in Japan [2]. There are two reasons for this. First, this trial did not use human biological specimens. Second, B-mode ultrasound is conducted as a routine examination for breast cancer diagnosis. However, public disclosure of information obtained in this study is required by all participating hospitals. When a patient refused to allow use of their clinical data, their data were not used.

### Centralized image interpretation committee

**Fig. 1:**
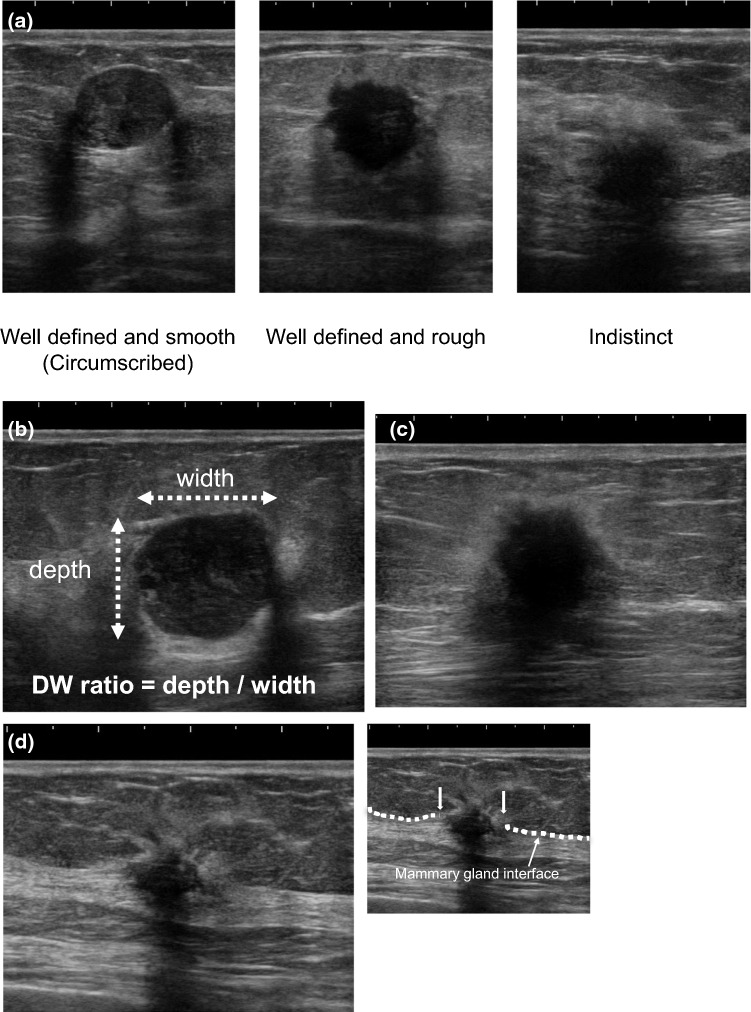
**a** Margin, **b** depth width ratio (DW ratio), **c** echogenic halo (echogenic rim), **d** interruption of mammary gland interface

Static B-mode digital images were evaluated by members of the centralized image interpretation committee comprised of 26 specialists with no knowledge of the clinical information (except for age) or the histopathological data. These 26 breast ultrasound specialists working in Japan included three radiologists, 19 breast surgeons, and four ultrasonographers. All were members of the Terminology and Diagnostic Criteria Committee of the JABTS. The 26 ultrasound specialists were divided into 13 pairs. Pairs of specialists evaluated each of the ultrasound images. If interpretation was difficult, the images were discussed by all members of the committee. The quality of liquid crystal image displays used for the centralized image interpretation was confirmed by TG18-QC pattern (American Association of Physicists in Medicine) [3]. The ultrasound findings and categories of each mass were reported by the centralized image interpretation committee. After evaluation of findings, such as shape, margin (Fig. 1a), internal echoes, posterior echoes, depth/width ratio (DW ratio, Fig. 1b), echogenic halo (echogenic rim, Fig. 1c), and interruption of the mammary gland interface (Fig. 1d), the B-mode category was determined by consensus.

### Japanese category

In Japan, we use Japanese categories; C1: normal, C2: benign, C3a: probably benign (observation is recommended), C3b: probably benign (biopsy is recommended), C4: suspicion of malignancy, C5: malignant [1]. Japanese categories differ from those of the Breast Imaging Reporting and Data System (BI-RADS) [4]. Japanese C3b corresponds to BI-RADS category 4A (biopsy is recommended) (Table 1).

**Table 1:** Correspondence between Japanese and BI-RADS categories

Japanese Category	BI-RADS Category
1	1
2	2
3a	3
3b	4A
4	4B, 4C
5	5

*BI-RADS* breast imaging reporting and data system

### The recall criteria for ultrasound breast cancer screening

**Fig. 2:**
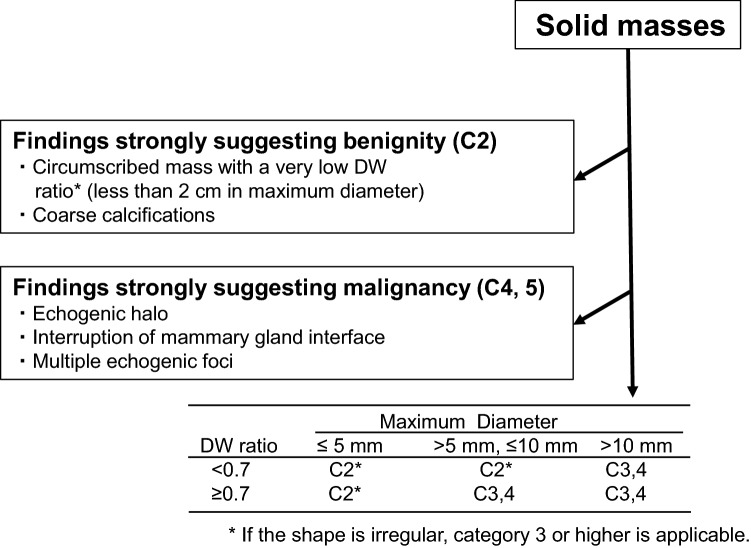
The recall criteria for solid masses (2004 version)

The recall criteria for ultrasound breast cancer screening include criteria pertaining to breast masses (simple cysts, complex cystic and solid masses) and breast non-mass lesions [1, 5]. In this study, we focused only on solid breast masses, and we developed a new diagnostic flowchart for solid breast masses based on the recall criteria. Figure 2 shows the recall criteria pertaining to solid masses (2004 version) [6]. Herein, we show a slightly simplified version of the original recall criteria to enhance understanding. The criteria are divided into three sections. The first section categorizes obviously benign masses (fibroadenomas) as C2. Typical fibroadenomas have an oval shape, circumscribed margin, diameter less than 2 cm, and a very low DW ratio. In this study, we defined “very low DW ratio” as less than 0.5. Typical calcified fibroadenomas are characterized by coarse calcifications. The second section categorizes obviously malignant or highly suspicious masses as C4 or C5. Typical malignant masses show an echogenic halo and/or interruption of the mammary gland interface [7]. Masses with a high possibility of malignancy show echogenic foci within the mass. In the third section, the remaining masses are categorized as C2, 3, or 4 according to the size and DW ratio. Since the third section of the recall criteria does not distinguish between C3a (observation) and C3b (biopsy), it cannot serve to determine whether biopsy or observation should be recommended. In 2014, an item pertaining to complicated cysts was added to the first section of the recall criteria [1, 5]. However, since the B-mode images were evaluated before 2014, this item was not included in the present study.

### Statistical analysis

Data collection and statistical analyses were conducted by the Clinical Research Data Center of Tohoku University Hospital. Statistical analyses were conducted using SAS Version 9.4 (SAS Institute, Inc., Cary, NC, USA). Univariate analysis was conducted using chi-square tests. Multivariate analysis was conducted using logistic regression.

### New diagnostic flowchart for solid masses

Based on the results of the statistical analysis, the Terminology and Diagnostic Criteria Committee of the JABTS endeavored to develop a new diagnostic flowchart. To facilitate user recollection of the essential points, we developed this new diagnostic flowchart based on the recall criteria already widely used in Japan.

### Verification of the usefulness of the new diagnostic flowchart for solid masses

The usefulness of our novel diagnostic flowchart for solid masses was evaluated by comparing the sensitivity and specificity determined by experts with those determined based on the new diagnostic flowchart.

We emphasize the importance of using a dataset from a different patient population, in addition to the one obtained in this study, to allow comparative evaluation of the usefulness of the new diagnostic flowchart. Therefore, in addition to data from the current study (JABTS BC-01), we employed the patient dataset from our JABTS BC-04 study [8]. The JABTS BC-04 study aimed to develop diagnostic criteria for color Doppler examination of solid masses in the breast, and was conducted from 2013 to 2017. The dataset from the JABTS BC-04 study included 839 malignant masses and 569 benign masses.

The datasets from the current (JABTS BC-01) study and the JABTS BC-04 study include findings and categories of solid masses determined by the centralized image interpretation committee. Categories of the new diagnostic flowchart were mechanically converted using the findings contained in the datasets. As a result, there were two categories for each mass; one determined by a specialist and the other based on the new diagnostic flowchart. We calculated and then compared sensitivity and specificity using these two categories. For statistical analyses of sensitivity and specificity, Japanese categories 2 and 3a (only observation is recommended) were considered to be negative, while categories 3b, 4, and 5 (biopsy is recommended) were taken to be positive.

### Study registration

The JABTS BC-01 study is registered with the University Hospital Medical Information Network, Japan (No. UMIN000007603).

## Results

**Fig. 3:**
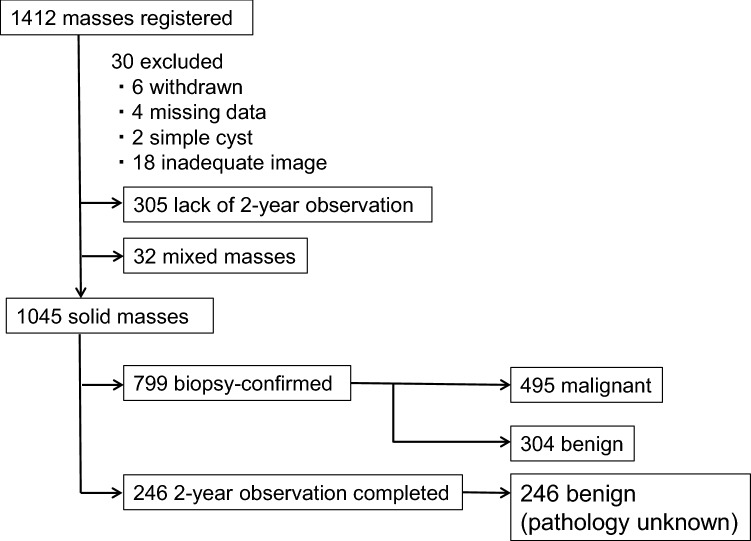
Flow diagram of registered masses

Between September 2009 and January 2010, 1412 ultrasound-visible breast masses were registered from 22 hospitals. Final enquiries regarding the histopathology and clinical observations were conducted in March 2014. Of the 1412 masses, six were excluded due to patient withdrawal, four due to missing data, two due to being simple cysts, and 18 due to being unevaluable by the centralized image interpretation committee because of inadequate image quality. Three hundred and five (55.3%) of 551 observational masses lacked 2-year observation results. Of the remaining 1077 masses, 1045 were solid. Since the number of mixed masses was only 32, we evaluated the 1045 solid masses (malignant: 495 (468 patients), benign: 550 (459 patients)) in this study (Fig. 3). Mean sizes of malignant and benign masses were 1.6 ± 0.78 cm (0.3–4.4) and 1.2 ± 0.71 cm (0.3–5.5), respectively. The ages of the 468 patients with malignant mases and the 459 patients with benign masses were 56.8 ± 12.5 years (mean ± standard deviation, range: 30–95) and 45.1 ± 11.9 years (13–76), respectively. The histopathological results of the 1045 masses are shown in Table 2. Biopsy was performed for 799 masses, of which 495 were malignant and 304 were benign. Invasive carcinoma of no special type accounted for 80% of malignant masses and ductal carcinoma in situ accounted for 10%.

**Table 2:** Histopathological results (*n* = 1045)

Malignant (*n* = 495)	
Ductal carcinoma in situ	47
Invasive carcinoma of no special type	402
Invasive lobular carcinoma	20
Mucinous carcinoma	18
Carcinoma with apocrine differentiation	2
Tubular carcinoma	2
Other (malignant)	4
Benign (*n* = 550)	
Fibroadenoma	115
Fibrocystic disease	71
Intraductal papilloma	28
Complicated cyst	25
Phyllodes tumor	8
Fibrosis	7
Ductal hyperplasia	5
Tubular adenoma	2
Other (benign)	43
Unknown (benign)^a^	246

^a^2-year observation completed

### Utility of the recall criteria as a diagnostic flowchart

We evaluated the usefulness of the recall criteria as a diagnostic flowchart. In the first and second sections of the recall criteria, 12.5% (69/550) of benign masses and 87.1% (431/495) of malignant masses were detected. Table 3 shows the malignancy rate in the first and second sections of the recall criteria. Regarding circumscribed masses with a very low DW ratio (diameter less than 2 cm), 98.4% (61/62) were benign, and 89% (8/9) of masses with coarse calcifications were benign. Furthermore, 97.5% (153/157) of masses with an echogenic halo, 90.8% (246/271) showing interruption of the mammary gland interface, and 59.3% (32/54) with multiple echogenic foci were malignant. Since the third section of the recall criteria for solid masses cannot be used in a diagnostic flowchart, we did not include it in the present evaluation.

**Table 3:** Malignancy rates in lesions found to be benign or malignant based on JABTS Recall criteria

Findings	Malignancy rate
Obvious benign findings (C2)	
Circumscribed mass with a very low DW ratio (diameter less than 2 cm)	1.6% (1/62)
Coarse calcification	11.1% (1/9)
Obvious malignant findings (C4, 5)	
Echogenic halo	97.5% (153/157)
Interruption of mammary gland interface	90.8% (246/271)
Multiple echogenic foci	59.3% (32/54)

### Frequencies of malignant and benign masses according to the ultrasound findings (Table 4)

**Table 4:** Frequency of malignant and benign masses according to US findings (n = 1045)

Findings	Number of cases	Malignant	Benign	% Malignant	% Benign
Shape					
Oval	313	33	280	10.5%	89.5%
Round	56	25	31	44.6%	55.4%
Lobulated	220	104	116	47.3%	52.7%
(2 or 3 undulations)	141	50	-91	35.5%	64.5%
(More than 4 undulations)	73	48	-25	65.8%	34.2%
Polygonal	47	22	25	46.8%	53.2%
Irregular	409	311	98	76.0%	24.0%
DW ratio					
< 0.7	616	206	410	33.4%	66.6%
(< 0.5)	(216)	(50)	(166)	(23.1%)	(76.9%)
(0.5–0.7)	(400)	(156)	(244)	(39.0%)	(61.0%)
≥ 0.7	429	289	140	67.4%	32.6%
Margin					
Circumscribed	337	30	307	8.9%	91.1%
Well-defined and rough	486	298	188	61.3%	38.7%
Indistinct	220	166	54	75.5%	24.5%
Obscure	2	1	1	50.0%	50.0%
Echogenic halo					
Present	157	153	4	97.5%	2.5%
Absent	888	342	546	38.5%	61.5%
Internal echoes					
Homogeneity					
Homogeneous	408	104	304	25.5%	74.5%
Heterogeneous	637	391	246	61.4%	38.6%
Echo level					
Anechoic	0	0	0	0.0%	0.0%
Hypoechoic	920	461	459	50.1%	49.9%
Isoechoic	119	31	88	26.1%	73.9%
Hyperechoic	6	3	3	50.0%	50.0%
Echogenic foci					
Present	97	73	24	75.3%	24.7%
Absent	948	422	526	44.5%	55.5%
Coarse calcifications					
Present	9	1	8	11.1%	88.9%
Absent	1036	494	542	47.7%	52.3%
Posterior echoes					
Accentuating	323	148	175	45.8%	54.2%
Not changing	551	214	337	38.8%	61.2%
Attenuating	168	132	36	78.6%	21.4%
Shadowing	3	1	2	33.3%	66.7%
Interruption of mammary gland interface					
Present	271	246	25	90.8%	9.2%
Absent	774	249	525	32.2%	67.8%

As to shape, 89.5% of oval masses were benign, and 76.0% of irregular masses were malignant. Regarding the DW ratio, 66.6% of the masses with a DW ratio less than 0.7 were benign, and 67.4% of those with a DW ratio of at least 0.7 were malignant. As to the margin, 91.1% of circumscribed masses were benign, and 75.5% with indistinct margins were malignant. Furthermore, 97.5% of the masses with an echogenic halo and 90.8% of those showing interruption of the mammary gland interface were malignant. Regarding echogenic foci, 75.3% of the masses with echogenic foci were malignant.

### Multivariate analysis

Multivariate analysis showed that shape, DW ratio, margin, echogenic halo, and interruption of the mammary gland interface were significant findings for distinguishing between benign and malignant masses (Table 5).

**Table 5:** Multivariate analysis of B-mode features and benign/malignant differential diagnosis (logistic regression)

Findings	Adjusted odds ratio	95% Confidence interval		*p*
Shape				
Round vs. oval	3.34	1.41	7.93	0.0062
Polygonal vs. oval	2.04	0.79	5.29	0.141
Irregular vs. oval	4.07	2.16	7.65	< .0001
Lobulated vs. oval	2.82	1.52	5.25	0.001
DW ratio				
≥ 0.7 vs < 0.7	2.64	1.7	4.1	< .0001
Margin				
Indistinct vs. circumscribed	3.38	1.62	7.04	0.0012
Well-defined and rough vs. circumscribed	5.3	3	9.39	< .0001
Echogenic halo				
Present vs. absent	9.82	2.79	34.57	0.0004
Internal echoes				
Heterogeneous vs. homogeneous	1.18	0.74	1.86	0.49
Echo level				
Severely hypoechoic vs. hypoechoic	0.6	0.15	2.39	0.47
Isoechoic vs. hypoechoic	0.58	0.3	1.12	0.1
Hyperechoic vs. hypoechoic	1.18	0.07	18.89	0.91
Echogenic foci or coarse calcification				
Echogenic foci vs. absent	1.74	0.89	3.42	0.11
Coarse calcification vs absent	0.18	0.03	1.11	0.06
Posterior echoes				
Accentuating vs. not changing	1.37	0.88	2.16	0.17
Attenuating vs. not changing	0.98	0.52	1.87	0.96
Shadowing vs. not changing	2.5	0.06	114.43	0.64
Interruption of mammary gland interface				
Present vs. absent	3.1	1.72	5.57	0.0002

### The new diagnostic flowchart for solid masses (Fig. 4)

**Fig. 4:**
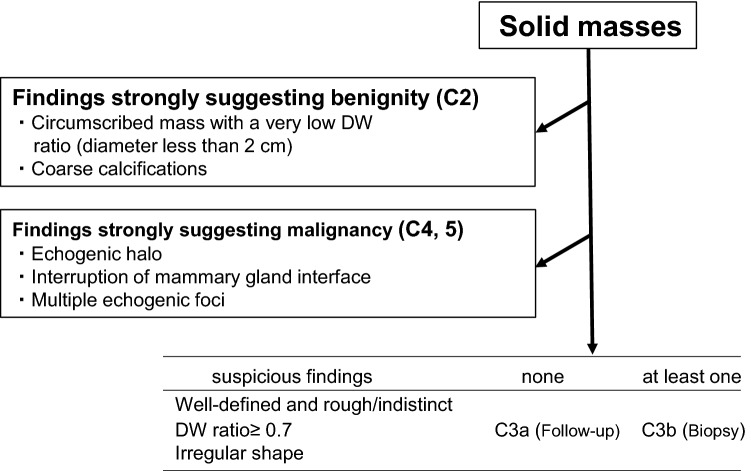
New diagnostic flowchart for solid masses

Using these results, the Terminology and Diagnostic Criteria Committee of the JABTS discussed and proposed a new diagnostic flowchart based on the recall criteria. Since the first and second sections of the recall criteria were demonstrated to be very useful, we applied them as the first and second sections of the new diagnostic flowchart. Then, we developed the third section of the new diagnostic flowchart. Of the five significant findings for distinguishing between benign and malignant masses by multivariate analysis, echogenic halo and interruption of the mammary gland interface were included in the second section. Therefore, we used shape, margin, and DW ratio in the third section. Among these parameters, those raising suspicion of malignancy were irregular shape, well-defined and rough/indistinct margin, and DW ratio ≥ 0.7 (Fig. 4). If none of these three suspicious findings is present, the category is determined to be 3a, and if at least one is present, the category is 3b. The committee proposed a new diagnostic flowchart for solid masses in May of 2015.

### Sensitivity and specificity of the new diagnostic flowchart

We calculated sensitivity and specificity using the datasets from the current study (JABTS BC-01) and the JABTS BC-04 study. Details of the BC-04 study dataset are shown in Table 6. With the current study dataset, sensitivity and specificity of the diagnostic flowchart were 0.97 and 0.45, respectively. The sensitivity and specificity based on the evaluations performed by the specialists were 0.96 and 0.54, respectively (Table 7). When we used the BC-04 study dataset, the respective sensitivity and specificity of the diagnostic flowchart were 0.95 and 0.45, while the corresponding sensitivity and specificity for the specialists were 0.98 and 0.38.

**Table 6:** BC-04 study dataset (*n* = 1408)

Number of masses	
Malignant: 839 (818 patients)	
Benign: 569 (547 patients)	
Age (mean ± standard deviation, range)	
Malignant (818 patients): 57.7 ± 13.7 (25–96)	
Benign (547 patients): 44.8 ± 12.8 (12–87)	
Mean sizes	
Malignant: 1.7 ± 0.84 cm (0.4–4.8)	
Benign: 1.5 ± 0.92 cm (0.3–4.8)	
Histopathological results	
Malignant (839)	
Ductal carcinoma in situ	53
Invasive carcinoma of no special type	716
Invasive lobular carcinoma	29
Mucinous carcinoma	22
Carcinoma with apocrine differentiation	5
Tubular carcinoma	1
Other (malignant)	18
Benign (569)	
Fibroadenoma	179
Fibrocystic disease	104
Intraductal papilloma	43
Complicated cyst	3
Phyllodes tumor	29
Ductal hyperplasia	6
Tubular adenoma	1
Other (benign)	29
Unknown (benign)^a^	175

^a^2-year observation completed

**Table 7:** Sensitivity and specificity using new diagnostic flowchart vs experienced specialists (C2, 3a vs. C3b, 4, 5)

	Datasets			
	Current study (JABTS BC-01) (*n* = 1045^a^)		JABTS BC-04 study (*n* = 1408^b^)	
	Sensitivity	Specificity	Sensitivity	Specificity
New diagnostic flowchart	0.97	0.45	0.95	0.45
Specialists	0.96	0.54	0.98	0.38

^a^Malignant: 495, benign: 550

^b^Malignant: 839, benign: 569

## Discussion

Ultrasonography is very useful for diagnosing breast cancer. There has been significant progress in distinguishing between malignant and benign masses using ultrasound since the early 1990s [9–12]. Several sonographic features based on shape, margin, and echo texture have been proposed for the diagnosis of breast masses [10, 13, 14]. At present, the BI-RADS classification is widely used globally [4]. However, BI-RADS does not include ultrasound diagnostic criteria. Several studies have attempted to develop ultrasound diagnostic criteria [15, 16], but diagnostic criteria have yet to be standardized.

In Japan, JABTS developed a diagnostic flowchart for breast masses in 2004 [6]. The diagnostic flowchart was developed after 4 years of discussions among experts. However, the flowchart was complex and therefore did not come into widespread use in Japan. On the other hand, the recall criteria for ultrasound breast cancer screening are very simple and widely used in Japan for ultrasound screening of breast cancer [5]. It would be optimal if the recall criteria could be used as a diagnostic flowchart. However, the recall criteria cannot be used to decide whether to recommend a biopsy or observation, and thus cannot be applied in diagnostic flowchart form. Therefore, we needed to develop a novel diagnostic flowchart. To encourage widespread use in Japan, we aimed to simplify the new diagnostic flowchart and to apply the recall criteria already widely used in Japan.

The recall criteria were proposed by JABTS in 2004 [6], and a revised version was published in 2016 [5]. Clearly or typically benign (fibroadenoma) and malignant masses are identified by applying the first and second portions of the criteria. Several reports have described fibroadenoma findings [17–20]. These findings are oval shape, smooth and well-circumscribed margin, and low DW ratio. The DW ratio was reportedly less than 0.7 in 86% of fibroadenomas and, furthermore, fibroadenomas usually stopped growing at a size of 2–3 cm [18]. According to the recall criteria, typical fibroadenomas are defined as oval circumscribed masses less than 2 cm in diameter, with a very low DW ratio. In this study, 1.6% (1/62) of masses judged to be typical fibroadenomas were ultimately found to be malignant.

The two typically used malignant findings are echogenic halo and interruption of the mammary gland interface. Echogenic halo has been reported to have high predictive value for malignancy by multiple research groups [13, 14, 16]. Interruption of the mammary gland interface was originally proposed by Konishi [7] in 1988. This finding has been used in Japan since the late twentieth century [21]. Our present study showed that this finding had high predictive value for malignancy. However, the predictive value of interruption of the mammary gland interface was slightly lower (90.8%) than that of echogenic halo (97.5%). Applying a combination of these two findings resulted in approximately half of breast cancers being interpreted as malignant. Features typical of malignancy (C4, 5) and of fibroadenoma (C2) in the recall criteria had high diagnostic utility (Table 2). Therefore, we decided to incorporate the first and second sections of the recall criteria into the new diagnostic flowchart.

We also examined the applicability of the third section of the new diagnostic flowchart. We used three findings raising suspicion of malignancy, which multivariate analysis had shown to be useful for distinguishing between benign and malignant breast masses. We advocate performing a biopsy if any of these three suspicious findings is identified in breast masses with neither clearly typical benign nor malignant findings. The sensitivity and specificity of the new diagnostic flowchart using the dataset from the current study were 0.97 and 0.45, respectively. The corresponding sensitivity and specificity of the current study for the specialists (centralized image interpretation committee) were 0.96 and 0.54. Furthermore, we examined the usefulness of the new diagnostic flowchart using our dataset from the BC-04 study [8]. The sensitivity and specificity of the new diagnostic flowchart using the BC-04 study dataset were 0.95 and 0.45, respectively. The sensitivity and specificity for the specialists (centralized image interpretation committee) examining the BC-04 study were 0.98 and 0.38. The specificity of the new flowchart was thus slightly inferior to that of the experts, but the sensitivity was higher. These results indicate that the new diagnostic flowchart is applicable, at a minimum, to diagnostic flowchart use for beginners. This flowchart is just a first step for beginners learning breast ultrasound. As they gain experience, beginners can progress to the intermediate and more advanced skill levels. This flowchart may even serve as a gateway allowing beginners to become experts in performing diagnostic ultrasound examinations of the breast.

We anticipate that this flowchart will become more sophisticated with ongoing revisions. As an example, parameters, such as age, elastographic findings, and color Doppler imaging, could potentially be incorporated into the flowchart. We hope that this flowchart will be useful not only to medical specialists but also to patients.

### Limitation

Our proposed diagnostic flowchart was developed based on expert-judged imaging findings data. This flowchart may not work well if the inter-observer agreement between the beginners and the experts in evaluating each finding is low. Therefore, there is a need for education for beginners using this flowchart.

## Conclusion

In this study, we developed a simple diagnostic flowchart for B-mode breast ultrasound. This flowchart is anticipated to be applicable to educating beginners learning breast ultrasound.
